# *In vivo* Microscopic Photoacoustic Spectroscopy for Non-Invasive Glucose Monitoring Invulnerable to Skin Secretion Products

**DOI:** 10.1038/s41598-018-19340-y

**Published:** 2018-01-18

**Authors:** Joo Yong Sim, Chang-Geun Ahn, Eun-Ju Jeong, Bong Kyu Kim

**Affiliations:** Bio-Medical IT Convergence Research Department, Electronics and Telecommunications Research Institute, Daejeon, 34129 Korea

## Abstract

Photoacoustic spectroscopy has been shown to be a promising tool for non-invasive blood glucose monitoring. However, the repeatability of such a method is susceptible to changes in skin condition, which is dependent on hand washing and drying due to the high absorption of infrared excitation light to the skin secretion products or water. In this paper, we present a method to meet the challenges of mid-infrared photoacoustic spectroscopy for non-invasive glucose monitoring. By obtaining the microscopic spatial information of skin during the spectroscopy measurement, the skin region where the infrared spectra is insensitive to skin condition can be locally selected, which enables reliable prediction of the blood glucose level from the photoacoustic spectroscopy signals. Our raster-scan imaging showed that the skin condition for *in vivo* spectroscopic glucose monitoring had significant inhomogeneities and large variability in the probing area where the signal was acquired. However, the selective localization of the probing led to a reduction in the effects of variability due to the skin secretion product. Looking forward, this technology has broader applications not only in continuous glucose monitoring for diabetic patient care, but in forensic science, the diagnosis of malfunctioning sweat pores, and the discrimination of tumors extracted via biopsy.

## Introduction

Diabetes mellitus is a growing global challenge as one in eleven presently suffers from diabetes worldwide, which is expected to nearly double within the next ten years^1,2^. When the blood glucose level is uncontrolled, diabetes can lead to complications of stroke, heart attack, and kidney failure, as well as lifetime consequences for a patient^3,4^. Therefore, the regular monitoring and subsequent immediate control of blood glucose level is significant. However, the current available methods are based on enzyme reactions that require a painful puncturing procedure of the fingertip with a lance to extract the blood invasively. This procedure not only makes diabetics unwilling to check their glucose level as frequently as doctors recommend but also exposes them to the risk of infection. To address these challenges, numerous efforts have been made toward alternative non-invasive approaches of glucose detection comparable to the currently available invasive techniques. Such efforts can be divided into two major categories: (i) optical methods and (ii) non-optical transdermal methods. Optical methods include Raman spectroscopy^5,6^, diffuse reflection spectroscopy^7,8^, thermal emission spectroscopy^9,10^, near-infrared absorption spectroscopy^11^, and mm-wave spectroscopy^12^. Non-optical methods include transdermal impedance spectroscopy^13^, and, sonophoresis and iontophoresis techniques^2^. In non-optical transdermal methods, physical energy (e.g., electrical, thermal, or ultrasound) is used to access interstitial fluids or blood, and the form of their implementations is typically easier to be miniaturized than that of optical methods. However, this method may change the skin properties and cause blistering, irritation, or erythema. In addition, the access of glucose level using these methods is typically indirect, and therefore, a multisensor system can be required to achieve reasonable accuracy^13,14^.

On the other hand, compared to non-optical techniques, optical methods are less prone to skin irritation and are highly selective for glucose sensing even in a complex matrix such as blood. One of the most well-known examples of optical methods is infrared spectroscopy, which induces rotational and vibrational transitions associated with chemical bonds within or between molecules. Raman spectroscopy is another prominent method used for non-invasive glucose monitoring based on the inelastic scattering of monochromatic light. The frequency of re-emitted light is shifted with respect to the original light, and gives information about rotational, vibrational, or low-frequency transitions^15,16^. It has sharper signal peaks, is less affected by water, and has fewer interfering spectra. However, this technique also suffers from some limitations such as interference with other biological molecules. For more detailed reviews on non-invasive glucose monitoring, see the references of^17–19^.

In recent years, the technique of photoacoustic spectroscopy (PAS) has been demonstrated for non-invasive glucose detection due to the higher sensitivity it offers relative to optical absorption spectroscopy^20–22^. In this method, a mid-infrared laser beam irradiated on skin produces a thermal expansion, thereby generating an acoustic wave which is affected by the sample’s absorption coefficient as well as the physical properties of the propagation medium, such as the thermal expansion coefficient and acoustic velocity^23^. In contrast to the near-infrared light, glucose has strong characteristic absorption in the infrared region because of the C-H-O stretching and bending vibration in the range of 800–1200 cm^−1^ ^24^. Despite the shallow penetration depth of mid-infrared light into skin (<100 μm), it still reaches the interstitial fluids found in layers such as in the stratum granulosum and stratum spinosum below the stratum corneum, i.e., the outer most layer of skin with a thickness of ~15–20 μm. The interstitial fluid is known not only to have shorter delay times (~5–15 min) in the increase of blood glucose level than other body fluids (e.g., tear, saliva, and urine), but also to represent a considerably clearer matrix than the blood mainly consisting of glucose, albumin, and trace of lactate^22^.

In these optical methods, the analysis of the received light signal is inherently complex because the glucose signal is often very weak and easily interferes with other signals from a variety of molecules in the blood and tissues. In addition, the variability and inhomogeneity of human skin poses a problem for the commercial application of *in vivo* optical monitoring. That is because the method is vulnerable to the inherent variability and inhomogeneity of human skin, which continually changes due to normal physiology. A major reason for the variability is that human skin secretes complex multiple products that can interfere with the mid-infrared light. The two major secretion products from skin are sebum (human skin oil) and sweat. Sebum is a waxy oily substance, whereas sweat consists mostly of water with solutes of lactic acid, glucose, urea, and minerals^25^. The minerals in sweat include sodium (0.8 g/l), potassium (0.2 g/l), calcium (16 μg/l), and magnesium (1.3 μg/l)^26^. Due to the strong absorption of mid-infrared light in the sweat, skin oil, and trace components, the spectrum of the mid-infrared photoacoustic spectroscopy can be affected severely by the amount and composition of the skin products^27^. The finger, palm, forearm, and earlobe are common locations for the skin spectroscopy measurement but we note that the exocrine glands of skin have varying distributions over much the body. The sebaceous glands, distributed over the entire human body except the palms and soles, play a role in producing sebum, which protects skin from microbial attacks and prevents transdermal water loss^28^. Sweat is secreted mainly from the eccrine gland to regulate body temperature in response to the sympathetic nervous system. The apocrine gland is restricted to certain parts of the skin such as the axilla and areola whereas the eccrine gland is distributed all over the body. Non-invasive glucose sensing is supposed to penetrate the skin layers that encompass the skin secretion gland and the microscopic structure of the skin tissues. Previous studies on non-invasive glucose measurements lacked a thorough investigation of skin secretion products and the microscopic structure of the skin tissues.

In this work, we present a method to address the challenges of non-invasive glucose measurements by increasing the reliability of detection on the micrometer scale. We obtained microscopic spatial information of the skin prior to the spectroscopic measurement using the same laser used for the spectroscopy. We found that the microscopic view of the skin on the fingertips with a mid-infrared laser was highly inhomogeneous because of the secretion from the eccrine sweat glands which strongly affected the mid-infrared spectra. However, the temporal and spatial variabilities were minimized by selecting intact locations where the secretion products were barely intrusive. For several decades, numerous efforts have been made to design non-invasive glucose detection methods, but the accuracy and repeatability are still below those of the invasive methods due to the skin secretion products. In addressing these long-standing challenges, our approach has immense potential in establishing such a technology in near future.

## Results

### Position Scanning Photoacoustic Imaging

**Figure 1:**
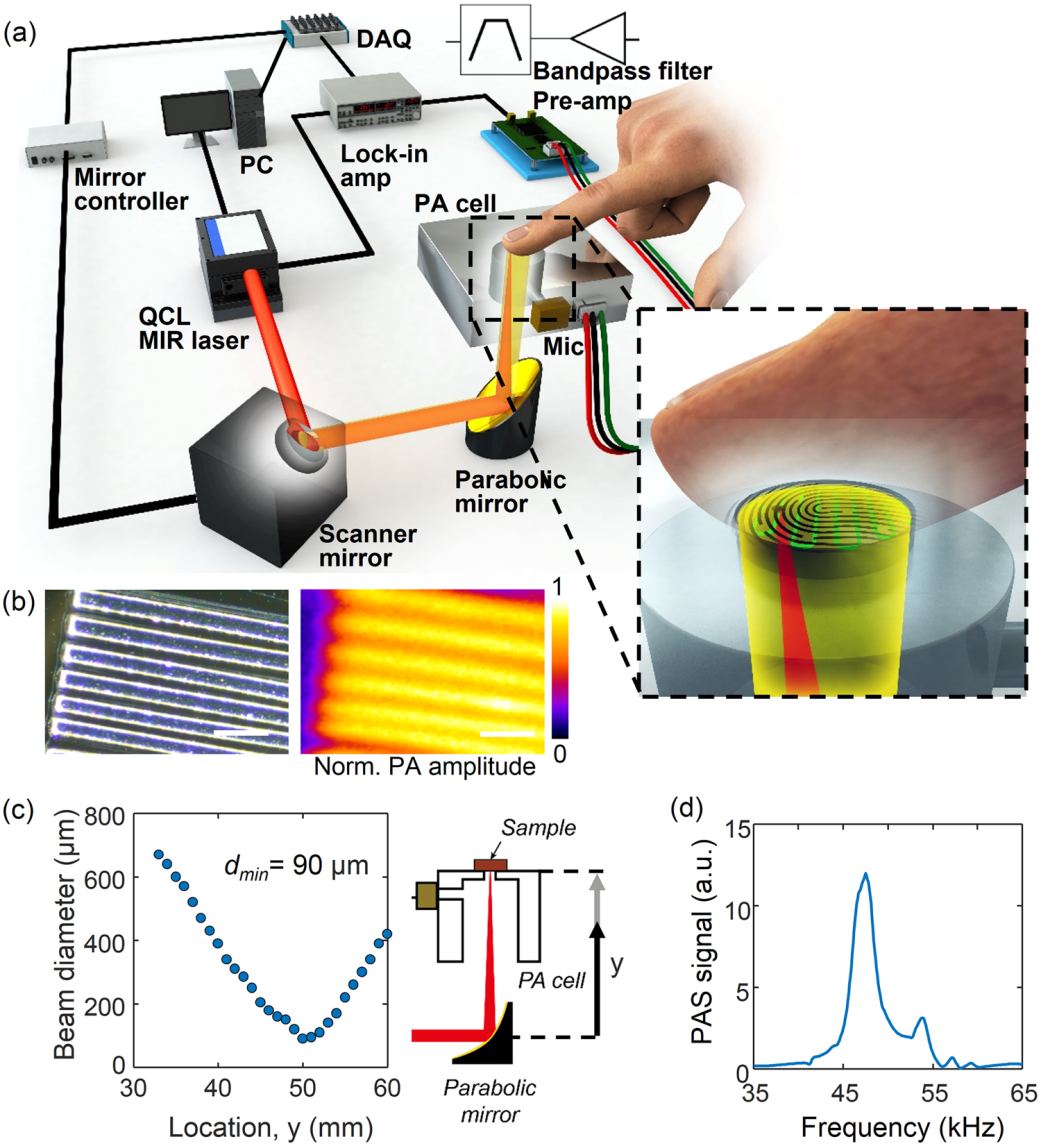
(**a**) Schematic drawing of the position scanning photoacoustic spectroscopy system. (**b**) Optical micrograph and photoacoustic image of SU-8 structures for resolution evaluation. (**c**) Beam diameter after reflection from the parabolic mirror, with a minimum of 90 μm at the focal distance of 50.4 mm from the parabolic mirror. (**d**) Photoacoustic spectrum in the frequency domain where the peak is located at 47.5 kHz with a reference carbon black tape sample. Scale bar is 250 µm.

Since the skin of our body constantly secretes sweat and human skin oil, these skin secretion products are a significant source of interference and noise. Therefore, it is important to investigate the effect of the sweat secretion on the *in vivo* skin photoacoustic spectroscopy for glucose monitoring. To obtain the spatial information of skin, we have developed a position scanning photoacoustic spectroscopy system. The setup for the mid infrared excitation of human skin *in vivo* and photoacoustic detection has been previously described^29,30^ and the modified version for the scanning photoacoustic spectroscopy is shown in Fig. 1. In our photoacoustic system, a high-energy laser beam irradiated on skin produced a thermal expansion, thereby generating an acoustic wave. The wave was acquired via a microphone, a phase-sensitive amplifier, and filters. The position scanning photoacoustic images of the skin was taken with a field of view of 1.3 mm by 1.3 mm and the skin is exposed to the detector in a 2.5 mm diameter. The laser beam was focused up to 90 μm in the beam diameter, near the focus at 50.4 mm as shown in Fig. 1(c). The resolution of photoacoustic imaging was expected to be 90 µm because the beam diameter was focused down to 90 µm. The resolution was experimentally evaluated using a specimen consisting of SU-8 line structures on a silicon wafer with varying line width and spacing. An optical micrograph and photoacoustic image of the SU-8 structures are shown in Fig. 1(b). The photoacoustic image confirmed that our system was able to resolve features as small as 90 µm. The size of the window was 30-by-30 pixels with a step size of 44 µm. The acoustic pulse of the skin generated by the absorption of the laser pulse was coupled to the air of the photoacoustic cell and by matching the repetition rate of the laser at 47.5 kHz, which is the frequency at which the resonance of the cell coincides with the resonance of the microphone, was amplified with a quality factor of 17 [Fig. 1(d)].

**Figure 2:**
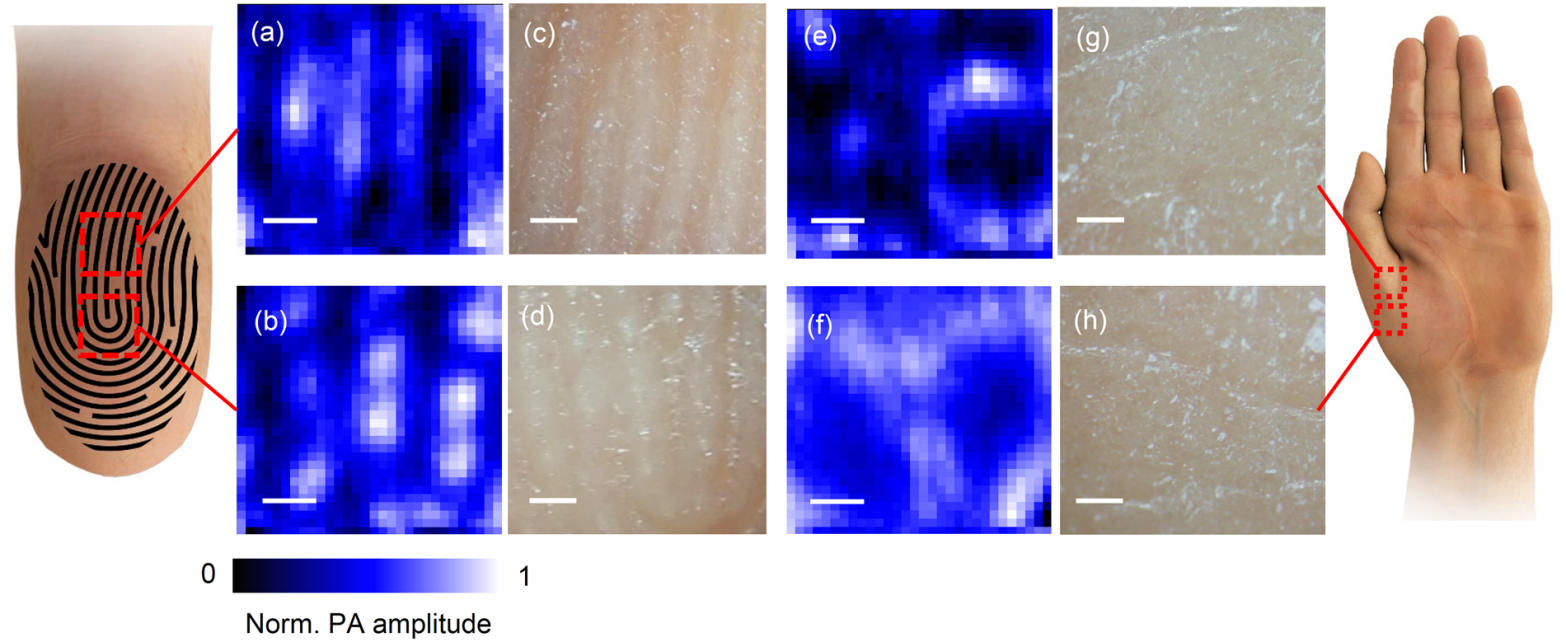
Images of the position scanning photoacoustic spectroscopy measured in two different regions of the fingertip; the part of the epidermal ridges aligned in a line (**a**) and the part where the epidermal ridges are bent into the U-shape (**b**). The optical micrographs of the corresponding fingertip regions are shown in (**c**) and (**d**). Images of the position scanning photoacoustic spectroscopy measured in the volar thenar are shown in (**e**) and (**f**), with the corresponding optical micrographs in (**g**) and (**h**). Scale bar is 250 µm.

Using the position scanning photoacoustic imaging system, we first investigated how the signal varied depending on the different locations of the skin. We obtained the position scanning photoacoustic images on multiple body locations of a volunteer including the fingertips, palm, and forearm. As shown in Fig. 2, a large inhomogeneity was found at the location where the fingertip (volar distal phalanx) was exposed to the detector. Depending on the probing locations of the fingertip, palm, and forearm, the patterns of the scanning images also varied to a large extent. Since the scanning location caused the skin inhomogeneity, variations would be introduced between subjects, as well as temporal changes even at the same location.

The microscopic images were taken in parallel at the same location where the photoacoustic images were taken [Fig. 2(c),(d),(g),(h)] The pattern of the scanning photoacoustic spectroscopy was well matched with the microscopic images at the corresponding site of the fingertips and the structural patterns of the friction ridges of the finger were reflected on the photoacoustic images [Fig. 2(a–d)]. However, instead of constructing the lines of the epidermal ridges, the pattern of the scanning photoacoustic spectroscopy had multiple discrete blobs along the epidermal ridges. The blobs of locally intense signals were observed along the top of the epidermal ridges across the entire set of images taken in the fingertip. The volar hypothenar of the palm displayed similar ridge structures coinciding with the friction ridges (data not shown) whereas the volar thenar showed rounded pore structures [Fig. 2(e)–(h)]. To visualize how the photoacoustic images co-register the optical microscopic photos, the two images of the finger index was merged varying the opacity of the photoacoustic images as shown in Supplementary Figure 2. We were unable to match the optical micrograph with the position scanning photoacoustic images of the thenar in which little friction ridges were present. We speculate that the patterns in the thenar resulted from the underlying structure which is not seen at the surface of skin.

### Spectra of Position Scanning Photoacoustic Imaging

**Figure 3:**
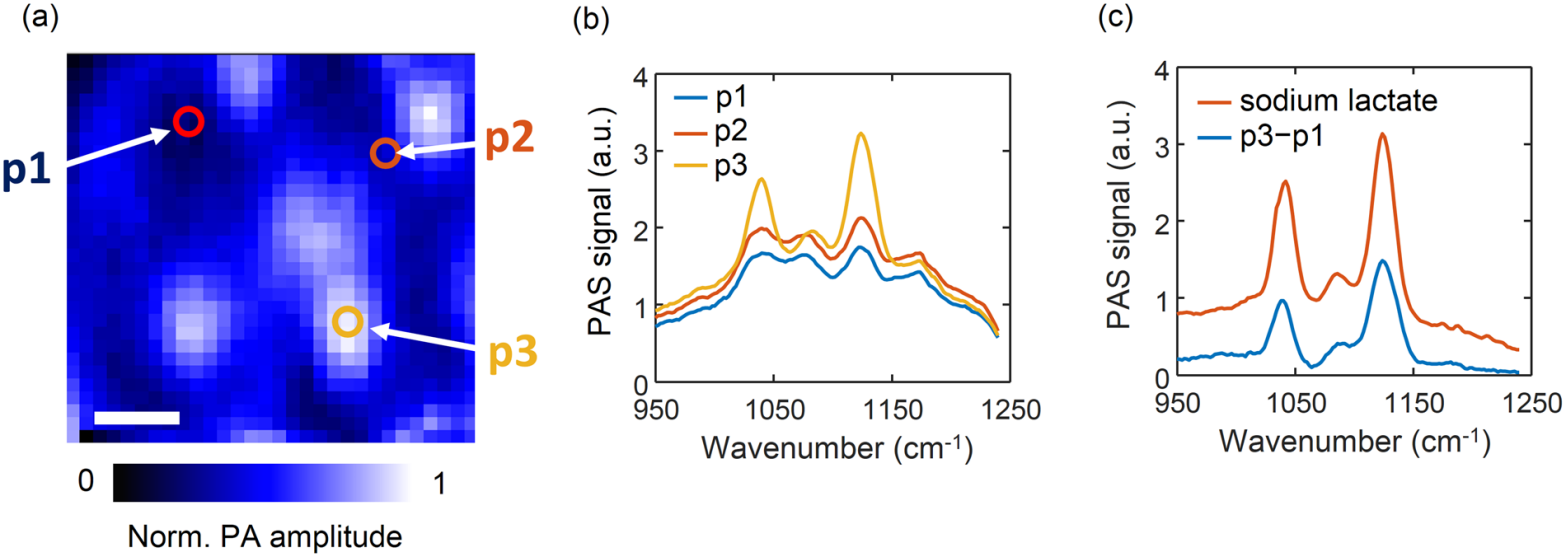
Spectra of different spots of a scanning photoacoustic spectroscopy image of skin and sodium lactate. (**a**) Three locations of the corresponding scanning image; p1 is in the dark region, p2 is the gray region between the bright blobs, and p3 is the bright region. (**b**) The wave number scan of the photoacoustic signal at the three different sites. (**c**) Spectrum of sodium lactate and the difference spectra of p1 from p3. Scale bar is 250 µm.

Wavenumber scanning of the mid infrared light was performed at multiple spots in the images: at the region of the locally intense blobs on the top of the ridges, at the dark region of the valley between ridges, and the gray region between the bright blobs on the top of the ridges. The different regions exhibited unique spectral patterns as shown in Fig. 3. At the bright blobs, large peaks were observed at 1070, 1105, and 1140 cm^−1^. The other locations had little or weak peaks at these wave numbers. This result implies that not only the signal intensities, but also the chemical compositions of the bright blobs differ from those of other locations.

On the fore side of the palm and fingers there are barely sebaceous glands but an abundance of eccrine sweat glands. The ducts of the sweat eccrine glands are usually open on the tops of the tiny lines on the forehand called epidermal ridges^31^. The sweat eccrine glands secrete the fluid products from the ducts in the skin primarily for thermoregulation. We hypothesized that the bright blobs in the position scanning images originated from the skin secretion products of the eccrine sweat or sebaceous glands. We therefore tested several different soluble analytes available in the sweat or sebum, including sodium lactate, calcium lactate, and triglycerides to see which chemical components contributed to the spectra. For comparison, we subtracted the spectrum of the dark region from that of the bright blob. The difference in the spectra allowed us to extract the chemical compositions of the secretion products from the epidermis. When we compared the difference spectra with those of the different analytes, the sodium lactate spectrum bore similarities with the spectrum of the bright blob with a comparable width and intensity (Fig. 3(c) and Supplementary Figure 2). We therefore concluded that the major component of the bright blob is sodium lactate.

### Effects of Temporal Skin Secretion on Photoacoustic Spectroscopy

The intense cleaning procedures of the skin before measurements would be beneficial to improve the day-by-day repeatability and reduce any spurious absorption effects of the dried-off layers of skin, as an optical measuring beam needs to penetrate the dried-off skin layers before reaching the interstitial fluid containing layers. In addition, cleaning procedures are a common practice before an invasive medical procedure, not limited to the photoacoustic measurement. However, the skin reconstitution by exocrine gland secretion in response to thorough hand washing could add further interference and increase measurement errors because the reconstitution response was found to be faster than the usual time required for a full glucose correlation test^27^.

**Figure 4:**
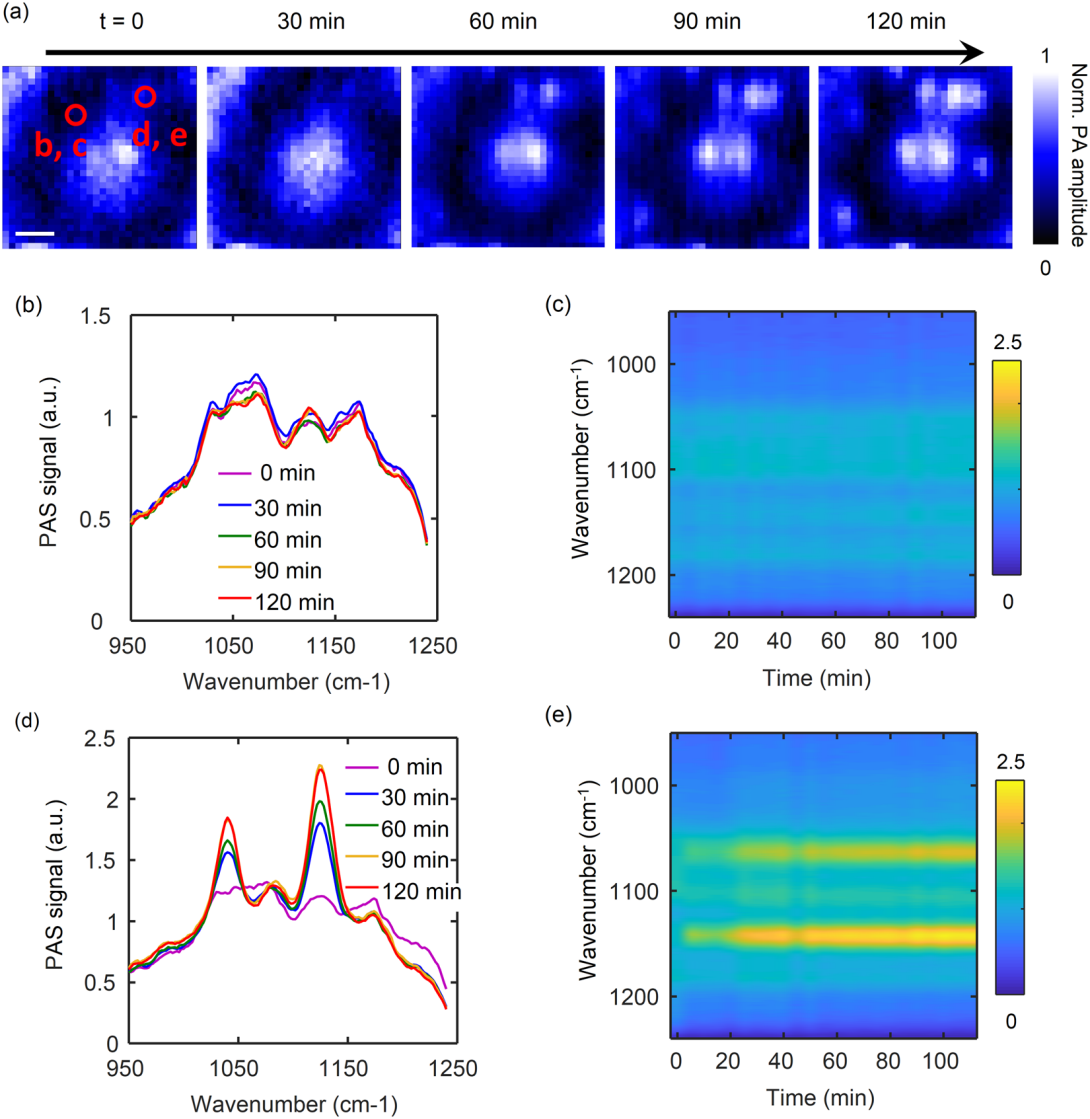
Images of the position scanning photoacoustic spectroscopy over time. (**a**) Images recorded for two hours in 30-min intervals. Scale bar is 250 µm. (**b**)–(**e**) Wavenumber scan over time at two different spots of a region of the index finger tip: (**b**) and (**c**) where there was no secretion, and (**d**) and (**e**) with secretion from an eccrine sweat gland. (**b**) and (**d**) 1D plots of the PAS spectra for each position. (**c**) and (**e**) Color coded representations of the spectral alterations observed for a volunteer.

To overcome these issues, we used the spatial information to detect the secretion of skin which created the spurious effects on the spectroscopy measurement. Prior to the measurement, the volunteers washed their hands thoroughly with soap and water, drying them off completely with nitrogen blow, before placing them on the measurement setup. While spectra were taken consecutively for two hours, the secretion of sweat was detected over time on spatially distinct locations as shown in Fig. 4(a). After hand washing the dark regions in the position scanning photoacoustic images remained steady. On contrary, some of the bright regions increased in the signal intensity and gained a similar shape as the bright blobs observed in the previous experiments without hand washing. This result proved that the detected bright blobs were associated with the sweat secretion from the eccrine glands.

Figure 4(b–e) shows the mid-infrared spectra obtained from the different locations. At the location without sweat secretion, the spectra stayed distinct from the spectra of the skin products and intact against the skin secretion [Fig. 4(b)]. Conversely, at the location with the sweat secretion, significant changes in the spectra were detected over time [Fig. 4(d)]. As expected, the peak location and width of the spectra here were also matched with the sweat spectra or the bright blob spectra previously measured. These results imply that the signals from the locations with the eccrine sweat glands strongly interfere with the glucose signals and dampen the mid-infrared light absorption of the glucose contained in the interstitial fluid. Notably, the intact region can be a promising location due to the reduction of the variations and increased repeatability of the measurements. Moreover, the pre-washing step right before the measurement was previously condemned because of the reconstitution of the skin^27^. However, if the measurement can be localized in the region with no skin secretion, the pre-washing step would be suitable for the glucose correlation test and preferred because it is invulnerable to the skin secretion product regardless of the reconstitution response.

The same spectral alternations due to skin secretion products are shown in a color-coded representation [Fig. 4(c),(e)]. At the non-secreting position, the signals were barely altered over time [Fig. 4(d)]. At the secreting position, the signals at 1070 cm^−1^ and 1140 cm^−1^ appeared in a few minutes after the start of the measurement with the washed hands, rose continuously, and saturated after about one hour [Fig. 4(e)]. It confirms that the position scanning photoacoustic spectroscopy can overcome the issues regarding the instant reconstitution response of skin to pre-washing as well as support the implementation of the glucose correlation test with photoacoustic spectroscopy after a pre-washing step.

Putting these data together, the dark area between the friction ridges was expected to prevent the effects of sebum and sweat. Our procedure for glucose measurement used the following protocol: (i) wash hands thoroughly and dry them off with nitrogen blow, (ii) obtain a repetition frequency response, (iii) acquire a 2D position scanning image and choose the location between the friction ridges, (iv) perform wavenumber scanning, and (v) measure the glucose level invasively. The 2D position scanning images displayed the friction ridges even immediately after washing hands. We initially located the probing position at the darkest point of the valley between the friction ridges. At a wavenumber of 1040 cm^−1^, the 2D position scanning images exhibited prominent signal differences by the skin secretion product. Additionally, if a dust particle resided on the skin, the images at the wavenumber allowed us to easily detect any particle. For these reasons, we chose a wavenumber of 1040 cm^−1^ of the QCL laser for 2D position scanning for the rest of the experiments.

### Glucose Correlation Test with Photoacoustic Spectroscopy

To assess the improvement by eliminating the interference of skin secretion, we conducted glucose correlation tests under the approval of the Institutional Review Board (IRB). We probed two locations per each glucose correlation procedure to evaluate the performance of photoacoustic non-invasive glucose monitoring when selectively probing the area: (i) the darkest point of the valley between the friction ridges and (ii) the brightest point on the friction ridges. During the glucose correlation test, the index finger of the subject was fixed to the photoacoustic cell so that the same probing position was maintained during the consecutive wavenumber scan. We ensured an identical probing location by acquiring the 2D position scanning at the end of the entire experiment and comparing it with the initial image.

One photoacoustic wavenumber scan in our system took 8.4 s. The total acquisition time of the 16 repetitive wavenumber scans of each location took 2.3 min, which corresponded to the reference measurements. The 2D position scanning added a time cost of 2 min to the glucose measurement. Provided that calibration is completed, a single measurement requires 4.3 min for glucose prediction. Further improvements in the measurement speed can be achieved by measuring at selected wavelengths instead of scanning the entire spectrum scan and reducing the size of the window for 2D position scanning. The imaging time may also be reduced by using an array of ultrasound transducers instead of a single acoustic sensor.

The pulse energy of the laser varies with different wavenumbers. Thus, one needs to normalize the photoacoustic spectra with the pulse energy variation curve for the wavenumber [Supplementary Figure 1(a)]. When we compared the prediction performance before and after normalization, it barely affected the glucose measurement results. This was because we were analyzing the relative difference of signals at each wavenumber. In addition, the single pulse energy of each wavenumber could fluctuate from its mean over time. To reduce the noise from the laser, we filtered the signal using a lock-in amplifier with a time constant of 30 ms, which took account of 1425 pulses. We additionally averaged 16 independent acquisitions to reduce possible deviations owing to laser pulse energy fluctuations. The publications of Pleitz *et al*. and Kottmann *et al*. reported methods to correct the laser power variation using a mercury cadmium telluride detector or a power meter, which may leave a room for a further improvement of our system^32,33^.

**Figure 5:**
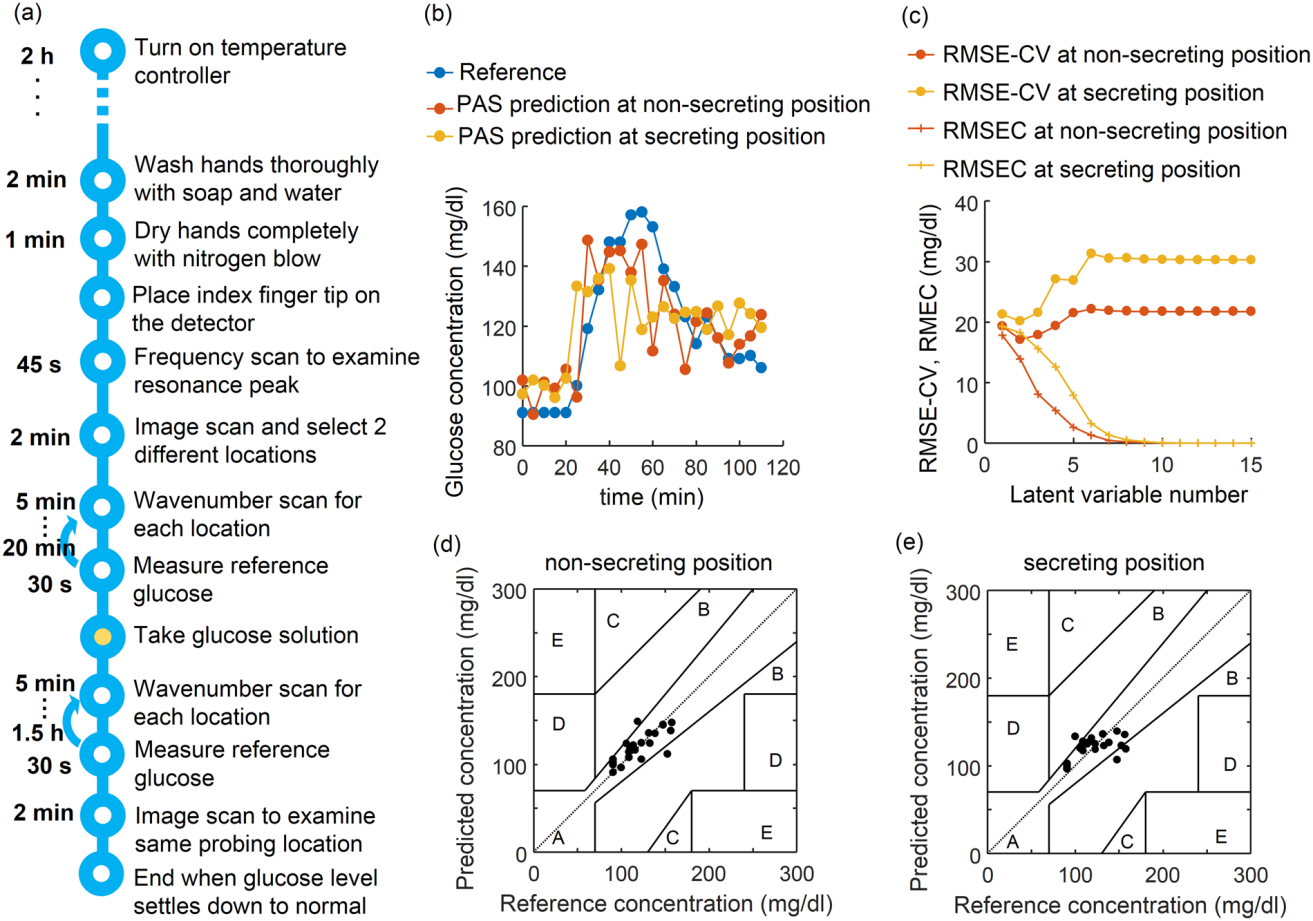
Performance of the glucose level prediction from the photoacoustic spectroscopy on two different spots with or without secretion from an eccrine sweat gland. (**a**) Procedure for glucose correlation test. (**b**) Non-invasively measured skin glucose with skin spectra recorded by photoacoustic spectroscopy on the two different spots of the index finger with (yellow) or without (red) secretion from an eccrine sweat gland of a volunteer over time compared with the reference glucose measured enzymatically after taking out blood (blue). (**c**) Changes in the root mean Square error of cross-validation and root mean square error of calibration with varying latent variable number in a PLSR analysis. (**d**) and (**e**) Correlation between measured blood glucose and predicted glucose from the spectra without (**e**) or with (**e**) the secretion from a sweat gland. The Clarke’s error grid is also shown.

To manipulate the blood glucose, an oral glucose tolerance test was performed by the intaking of glucose dissolved in water, and was monitored by the non-invasive infrared and invasive enzymatic measurements in parallel. After each wavenumber scan, the reference blood glucose level was measured. The detailed procedure for the glucose correlation test is shown in Fig. 5(a). To predict the glucose level from the photoacoustic spectrum, we used a Partial Least Square Regression (PLSR) algorithm and cross-validation. For details, see *Methods* and Supplementary Figure 5 for the algorithm. Figure 5 illustrates the prediction of the blood glucose level from the photoacoustic measurements of the skin on the index finger of a volunteer. Figure 5(c) is the glucose correlation test results over time calculated from the PLSR cross-validation of the data at both the non-secreting and secreting positions, and the reference glucose level. The non-invasive measurement closely followed the invasive reference measurements and no significant delay was observed. In particular, from the spectra at the position of non-secretion from an eccrine sweat gland the performance of the glucose level prediction was better than that of the prediction at the region near an eccrine sweat gland secretion. This time course result showed that the photoacoustic infrared spectra of intestinal fluid can be used to reliably determine the glucose level in the blood. Figure 5(c) illustrates the RMSE-CV (root mean square error of cross-validation) and RMSEC (root mean square error of calibration) by varying the number of the latent variable number used for the prediction. RMSEC monotonically decreases with the number of latent variables. However, RMSEC cannot fully reflect the model performance of prediction, and therefore RMSE-CV is more appropriate for evaluating the model performance of prediction. In both cases of non-secreting and secreting positions, the RMSE-CV decreased until the second latent variable was included and started to increase thereafter, so that we chose two latent variables for the prediction. In determining the agreement of the method with the clinical relevance of the errors, a Clarke’s grid was employed [Fig. 5(d),(e)] which is a standard measure to determine the accuracy of blood glucose measuring methods. It defines a region of sufficient accuracy (within 20% of the reference sensor, zone A) and a region of low but clinically acceptable accuracy without inappropriate treatment of the patient (zone B). The results in zones C, D, and E are potentially dangerous and are therefore clinically significant errors^34^. In addition, the Mean Absolute Relative Deviation (MARD) and the Mean Absolute Difference (MAD) of the cross-validated measurement data sets are also computed to compare system performance^35^. A MARD of 9.94% and MAD of 8.27 mg/dl were obtained for the non-secreting position. These values were 30% lower than the MARD of 14.67% and MAD of 11.98 mg/dl obtained from the secreting position. The standard deviations were, respectively, 9.97% and 7.5 mg/dl of MARD and MAD for the non-secreting position, and 11.12% and 7.99 mg/dl of MARD and MAD for the secreting position.

**Figure 6:**
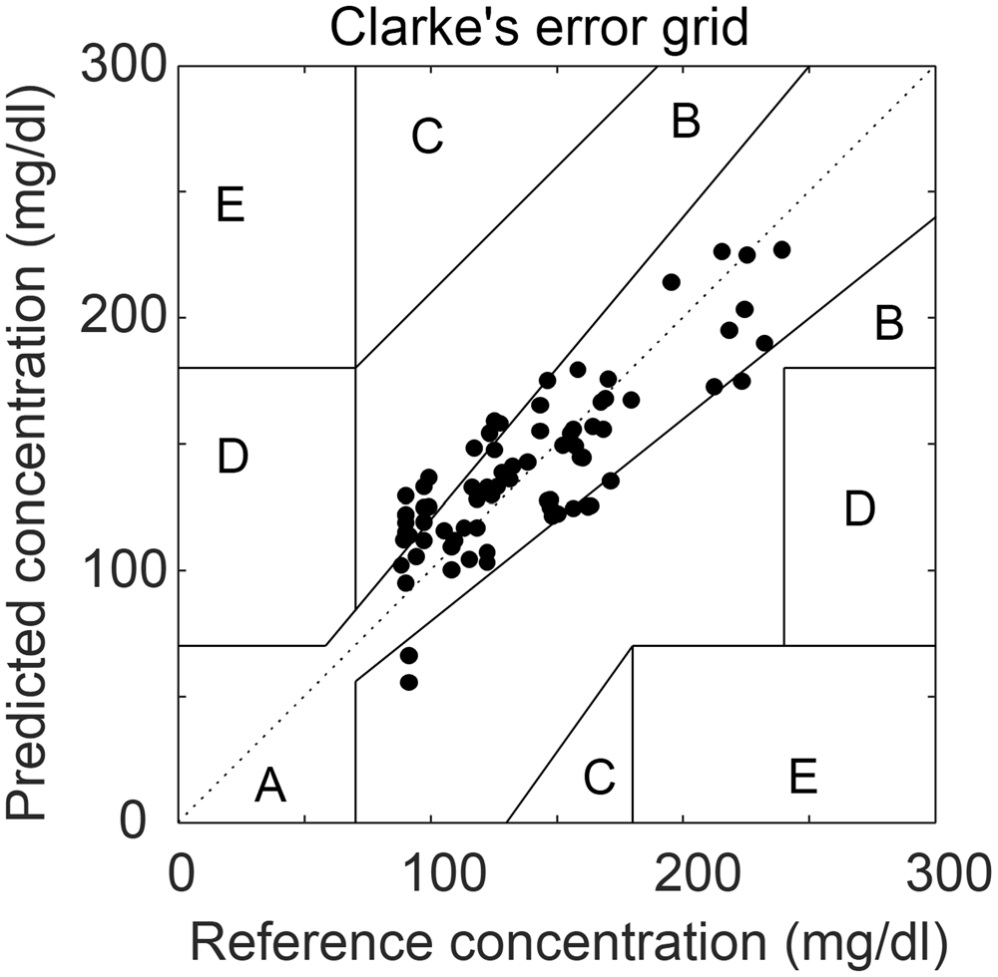
The pooled glucose correlation test from a healthy subject and a diabetic subject, and 70% of the measurements fell into Zone A.

These results confirmed that the infrared measurement on the non-secreting position produced a better prediction than that on the secreting position. By selectively probing the non-secreting position spectra of the fingertip, we continued conducting the five different experiments from both a healthy and a diabetic subject. As a result, PLSR-CV predictions from 76 measurements were acquired and 70% of measurements fell into zone A of the Clarke’s grid and 30% into zone B (Fig. 6). No measurement pairs fell in zones C, D, or E of the error grid. The MAD and MARD of the experiment resulted in 18.51 mg/dl (±12.35 mg/dl) and 14.4% (±10.5%), respectively, for the pooled data from the five independent experiments with a healthy and a diabetic subject.

## Discussion

In this study, we found that the microscopic structure and secretion products of skin tissues had a significant impact on non-invasive glucose measurements in mid-infrared photoacoustic spectroscopy. To the best of our knowledge, this is the first report on the microscopic position scanning of spectral signals for non-invasive glucose measurements. Our method was an improvement over most of the previously reported methods^13,14,36–39^, and performed worse than several previous approaches such as Raman Spectroscopy^15^ and photoacoustic spectroscopy^40^. However, skin secretion products and the microscopic structure of the skin tissues were not explored in previous invasive-glucose-sensing studies. Regardless of whether optical or non-optical means are employed, the spatial heterogeneity and temporal variability of the photoacoustic signal can contribute significant data variations. Therefore, our results provide unique insight into reducing the variations in day-to-day and person-to-person, which is one of the biggest obstacles in the field of non-invasive glucose monitoring. The spatial heterogeneity and temporal variability of the photoacoustic signal that we observed in the micrometer scale can also considerably contribute to data variation in the other non-invasive glucose measurement methods. The significant merit of our approach is that it is applicable to other photoacoustic glucose sensing approaches. The applicability can be extended to other modalities of non-invasive glucose sensors.

Non-invasive glucose monitoring has to meet several requirements for commercialization, such as the minimum number of calibration, accuracy, and day-to-day repeatability^2^. Our approach here provides a promising tool to achieve day-to-day repeatability, as the skin condition and the skin’s exocrine activity can be controlled. We found that after thorough hand washing, the valley between the epidermal ridges were intact from the skin secretion and therefore more robust than the region where the eccrine sweat pores are located on the top of the epidermal ridges. Recently, depth profiling of skin using photothermal deflection spectroscopy was introduced to address the challenges of individual skin and day-to-day variations^41^. Combining the spectral raster scan of mid-infrared light with the depth sensitive spectral information can be a powerful tool, enabling the correction for these in order to obtain a more precise glucose determination. As for commercialization using the current form of our system, owing to its high cost, a hospital is appropriate for those who can purchase the entire system. The majority of the cost belongs to the semiconductor mid-infrared laser capable of varying wavelengths. The scale of economy in manufacturing of the mid-infrared laser may reduce the cost of the laser, and additional efforts can be made by selectively choosing a few or less, fixed wavelengths of the laser.

An optimal location for a non-invasive glucose measurement needs to provide good correlation and no time lag with blood glucose, and allow ease of access for skin exposure to the infrared beam during the measurements. In addition, the probing site should be sufficiently circulated, quickly equilibrate the glucose of interstitial fluid with blood, and have a modest thickness of the stratum corneum. Bauer *et al*. reported that among four locations (the lower part of the arm, the hypothenar, the index finger, and the thumb), the index finger and the thumb appeared to be the best sites for measurement.^27^ In this study, the volar distal phalanx for the probing site was chosen to be probed for glucose detection because it satisfied the requirements and was the exact location for parallel reference measurements of blood glucose with pricking to take out blood. Putting our data together, additional thoughts on the gland density can be given, as the density of eccrine sweat glands in volar distal phalanx is relatively high. For example the gland density was reported as 350 glands/cm^2^ in the volar distal phalanx of the finger which is denser compared with the 126 glands/cm^2^ in the dorsal distal phalanx of finger, 108 glands/cm^2^ in the dorsal forearm, or 155 glands/cm^2^ in the nose^42^. Therefore, when probing the volar distal phalanx, care must be taken for the sweat secretion. Since the gland density was denser in the volar distal phalanx, exploring other probing positions would be of interest in future studies. Differences in skin composition and structures throughout the body also hold immense potential when investigated by our system. The microscopic photoacoustic images obtained in this study reflect not only the surface of the skin but also the underlying structures because the mid-infrared light penetrates the epidermis under the stratum cornea, as implied in the microscopic photoacoustic images of the volar thenar (inner palm) [Fig. 2(e)–(h)**]**.

In photoacoustic spectroscopic imaging, the photoacoustic signal can create a chemical image gathered from several other surfaces which is a complex mixture of natural latent secretions, providing the chemical compositions of the substances. For example, a chemical image of a fingerprint collected through tape lifting from different surfaces can provide a potential fingerprint chemical composition^43^. This special chemical imaging approach clinically allows for the diagnosis of the malfunction of sweat pores by accurately mapping active sweat pores as a function of spatial position without using added labels or dyes^31^ as well as for a component analysis of the sweat and skin conditions. Moreover, it can also be used to discriminate tumors extracted via biopsy including skin cancers^44–46^. By directly analyzing the components of the fluids using the photoacoustic spectroscopic imaging, various studies of microfluidic processes can also be conducted, ranging from reactions to the separation of large volumes for chemical and biological analysis^47,48^.

## Methods

### Scanning Photoacoustic Spectroscopy Setup

In our photoacoustic system, the acoustic pulse of the skin generated by the absorption of the laser pulse was coupled to the air of a photoacoustic cell before entering a microphone. Because air was used as an acoustic coupling medium, the air temperature was likely to affect the measurement. To suppress the source of signal variability, we maintained a constant temperature of 24 °C throughout the measurements. Acoustic coupling with air instead of ultrasound gel or oil was used owing to the shallow penetration depth of the mid-infrared laser. The shallow penetration depth generated a signal at the shallow skin layer, but the use of ultrasound gel or a similar coupling medium could cause absorption of the laser light and no impact on the skin tissues at all. A signal from a shallow depth (<100 µm) may create difficulties in locating an acoustic detector because the detector is supposed to be located close to the region of incidence of the laser light in the perpendicular direction of the acoustic wave propagation. In order to overcome these challenges, researchers reported on an optical-acoustic beam combiner or photoacoustic cavity to guide the ultrasound to the acoustic detector^22,49,50^. We chose the latter case, as reported previously^21,29,30,32^.

Figure 1 and Supplementary Figure 1 show a schematic drawing of the system and its basic characteristics. The system consisted of a mid-infrared laser source (an external-cavity quantum-cascade laser or EC-QCL, Über Tuner 9, Daylight Solutions, Inc., San Diego, CA, USA), a two-axis fast steering mirror system (FSM-300-02, Newport Corporation, Irvine, CA, USA), a photoacoustic cell, and a lock-in amplifier (SR844, Stanford Research Systems, Inc., Sunnyvale, CA, USA). The EC-QCL served as a laser source, which can tune the wavenumber from 900 cm^−1^ to 1250 cm^−1^ and modulate the repetition frequency ranging from 0.1 kHz to 100 kHz with the maximum pulse width of 500 ns. The laser was capable of irradiating with the maximum peak power of 150 mW with the beam size of 2 mm in diameter. The reference spectrum of the laser power was obtained using a power detector and monitor (XLP12-3S-H2 and MAESTRO, Gentec Electro-Optics, Quebec, Canada) at an operation repetition frequency of 47.5 kHz and a pulse width of 500 ns and is shown in Supplementary Figure 1(a). The maximum average laser power was 2.1 mW with single-pulse energy of 44.2 nJ (single-pulse power of 88.4 mW) at a wavenumber of 1000 cm^−1^ and a pulse duration of 500 ns. The pulse duration of the laser was set at 500 ns for all the experiments. This pulse energy varies with the wavenumber in the same ratio as the average power as shown in Supplementary Figure 1(a).

To achieve wavenumber scanning, the laser was programmed to scan across a selected tuning range of 950 cm^−1^ to 1240 cm^−1^ in forward- and reverse-scan modes as fast as 16.8 s per round sweep. The laser scan sampling density was 1.38 cm^−1^/each data point. The number of wavenumbers for spectrum measurement was 210 for a single one-way scan. The lock-in amplifier was set at a time constant of 30 ms with a filter slope of 12 dB/octave. To acquire the spatial information of human skin at the desired wavelengths of EC-QCL, we scanned a focused mid infrared laser light using the two-axis motorized mirror in X and Y, reflected by a right-angle off-axis parabolic mirror (MPD019-M01, Thorlab, Inc., Newton, NJ, USA) with a 50.8 mm focal length. During 2D position scanning, the wavenumber of the QCL laser was set to 1040 cm^−1^. The microscopic images were taken in parallel at the same location where the photoacoustic images were acquired using a stereo microscope (SZ61TR, Olympus Inc. Tokyo, Japan) with an interchangeable-lens camera (A6000, Sony). The diameter of the main cavity of the photoacoustic cell was set to 8.25 mm which resulted in the largest signal to noise ratio when its resonant peak matched the resonance of the ultrasound transducer (SPM0404, Knowles Inc.)^29^.

### Setup for *in Vivo* Skin Studies

An index finger tip was selected as the location to be probed^27^. One scan required 8.4 s and represented the tuning of the QCL over its complete emission spectrum in the range of 950–1240 cm^−1^. In parallel, the conventional invasive blood glucose measurements were conducted every 5 min with a blood glucose meter (Accu-Chek Performa II, Roche Diagnostics GmbH, Germany) that had 93 and 68% accuracies within a 10 and 5% error grid for blood glucose concentrations larger than 75 mg/dl. The IRB of the Ministry of Health and Welfare of Republic of Korea approved this study and written informed consent forms were obtained from all study participants. All experiments were performed in accordance with relevant guidelines and regulations. The invasive blood glucose measurements were conducted for the reference glucose level using the Accu-Chek Performa II and Accu-Chek Softclix lancing device (Roche Diagnostics GmbH, Germany) from the index finger that was not being examined. It took 5 s to complete one measurement after pricking a fingertip and infuse blood to a test strip. To induce the change in the blood glucose levels of the healthy and patient volunteers, a 300-ml solution of water containing 75 g of glucose was consumed 20 min after the start of the photoacoustic measurements, which corresponds to the standard condition of an oral glucose tolerance test. Prior to placing the index finger tip on the photoacoustic cell, the subject washed his/her hands thoroughly with soap and water, drying them completely with nitrogen blow. The entire procedure of the glucose correlation test is summarized in Fig. 5(a). To test the several different soluble analytes available in sweat including lactate, sodium, and calcium, we prepared sodium lactate, calcium lactate, sodium chloride, and calcium chloride in aqueous solutions.

### Data Analysis

In each glucose correlation test, we obtained 23 pairs of photoacoustic spectra and the corresponding blood glucose levels. The acquired spectrum data from the glucose correlation test consisted of multiple measurements for individual wavenumbers. Therefore, the analysis requires multivariate statistics to find a dependence between the spectrum data and glucose level with a number of parameters. In addition, the prediction of glucose level is a continuous variable, which requires a regression analysis to create a mapping between two blocks of data: (i) spectrum data X and (ii) glucose level Y [in Supplementary Figure 5(a–c)]. One of the available regression models is multiple linear regression. However, because the size of the sampled data was smaller than the parameter of independent variables at the wavenumbers, it can easily turn into an ill-conditioned problem. Thus, it is necessary to reduce the number of variables. To reduce the number of parameters, principal component analysis (PCA) can be used by finding principal components in a rotated orthogonal space which explains the majority of the variances in the measured spectrum data block. After reducing the number of parameters using PCA, a regression model can be used to obtain glucose predictions in the reduced input space. This procedure is called Principal Component Regression (PCR). On the other hand, PLSR also rotates the coordinate system of the data space and computes a new component called a latent variable, but it maximizes both the variance and the correlation between the dependent variables of the measured spectrum data block X and the reference glucose level Y. We compared the predictions by PCR and PLSR, and the result is shown in Supplementary Figure 5 (d–f). Each regression model of PCR and PLSR resulted in a MARD of 8.95 and 8.67, respectively. PLSR resulted in a slightly improved prediction; therefore, we used PLSR for the rest of the analyses. A PLSR model was constructed after data preprocessing through mean centering, and a leave-one-out cross-validation was performed in each glucose relationship test to obtain RMSE-CV. The number of latent variables were selected, such that the value was changed until RMSE-CV showed the minimum value and optimized between the distributed modeling and the over-fitting. For the PLSR analysis, Matlab (Matlab 2016a, Mathworks, Inc., Natick, MA, USA) and PLS Toolbox (PLS_Toolbox R8.2.1, Eigenvector Research Inc., Manson, Wash., USA) were employed.

## Electronic supplementary material

Supplementary Figures
