# Supplementary material for: In vivo Microscopic Photoacoustic Spectroscopy for Non-Invasive Glucose Monitoring Invulnerable to Skin Secretion Products
Source: Sci Rep. 2018 Jan 18;8:1059. doi: 10.1038/s41598-018-19340-y (PMC5773698; doi:10.1038/s41598-018-19340-y)
Supplement: Supplementary file 1 — Supplementary Figures [file 41598_2018_19340_MOESM1_ESM.pdf]

Supplementary Information for

In Vivo Microscopic Photoacoustic Spectroscopy for Non-Invasive Glucose Monitoring Invulnerable to Skin Secretion Products

Joo Yong Sim[1], *Chang-Geun Ahn*[1], *Eun-Ju Jeong*[1], *and Bong Kyu Kim*[1,*]
[1] Bio-Medical IT Convergence Research Department, Electronics and Telecommunications Research Institute, Daejeon 34129, Korea

*Corresponding Author: E-mail: bongkim@etri.re.kr

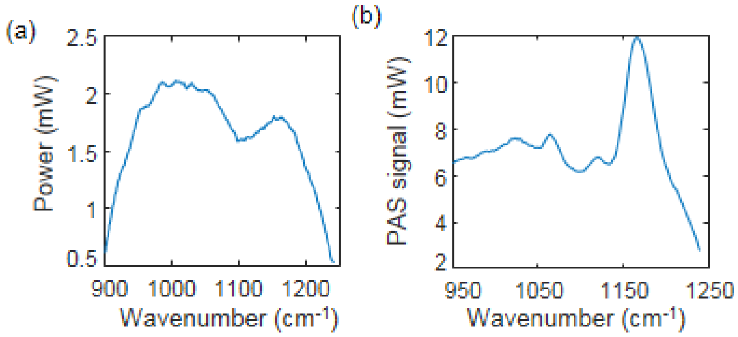

Figure S1. (a) Power of the QCL mid infrared laser varying its wave number from 900 to 1240 cm^{-1} at the operation repetition frequency of 47.5 kHz. (b) Photoascoustic spectrum of carbon black tape as a reference sample.

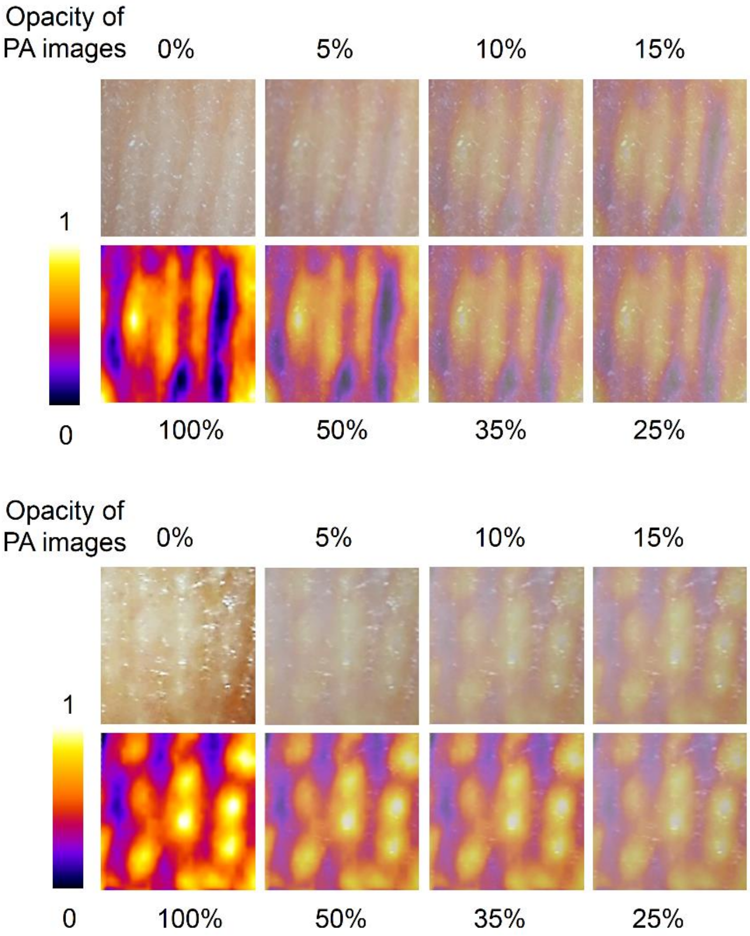

Figure S2. Merged two images of the finger index varying the opacity of PA images from 0%, 5%, 10% , 15%, 25%, 35% and 50% to 100% to visualize how the PA images co-register the optical microscopic photos.

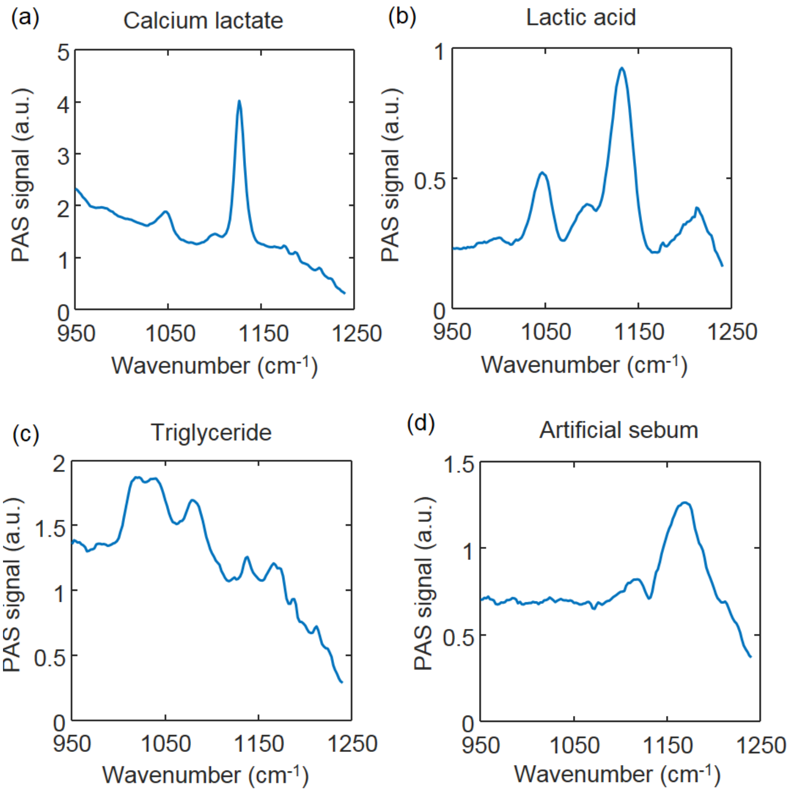

Figure S3. Mid infrared spectra of the sweat and the sebum components acquired by the photoacoustic spectroscopy system. The spectra of calcium lactate (a), lactic acid (b), tryglyceride (c), and artificial sebum (d).

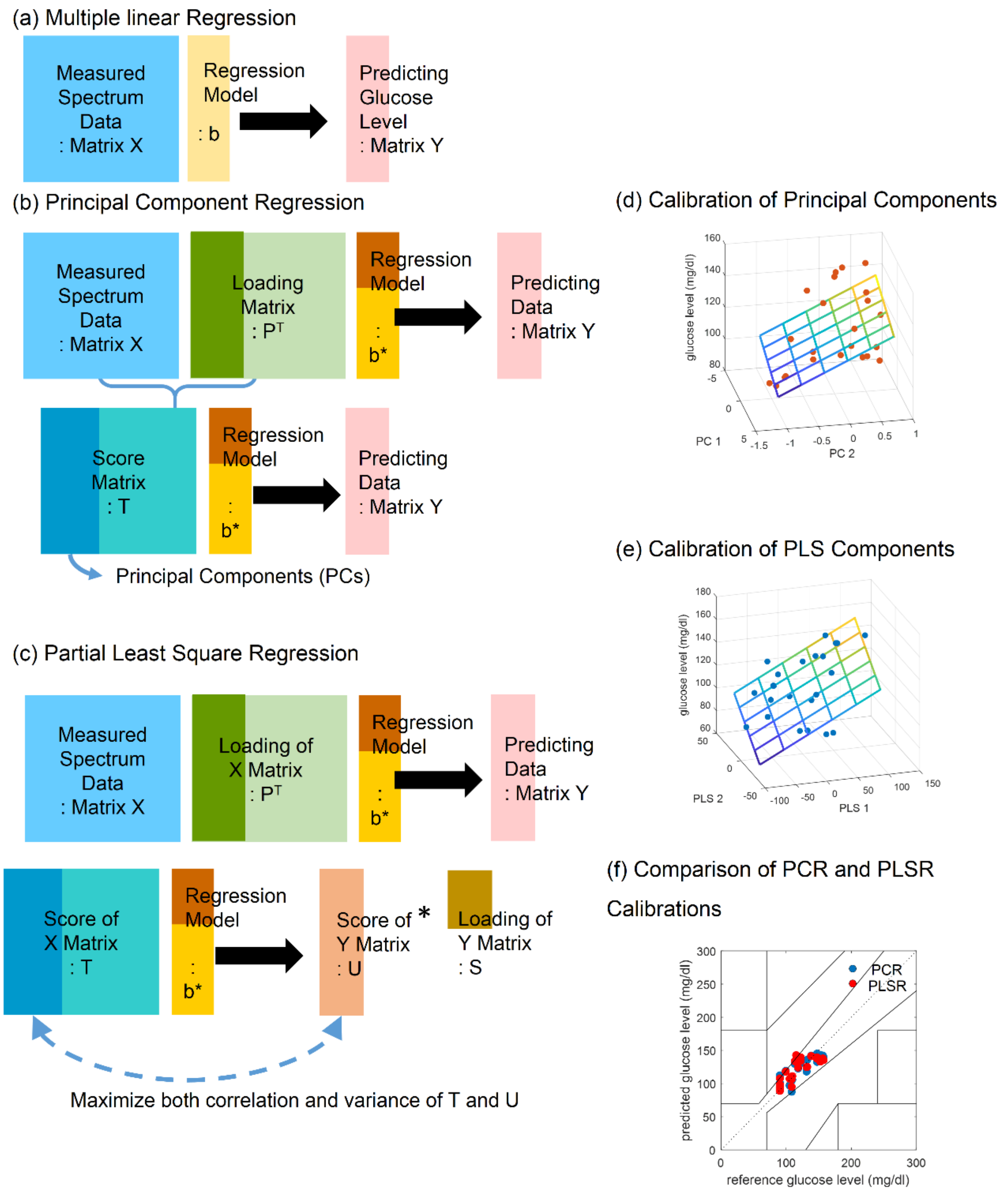

Figure S4. Multivariate regression models. The acquired spectrum data from the glucose correlation test was multiple measurements for 210 individual wavenumbers, which requires to analyze the data using multivariate statistics. To find a dependence between the spectrum data and glucose level, we need a regression analysis to create a mapping between two blocks of data: (i) spectrum data **X** and (ii) glucose level **Y**. (a) Multiple linear regression is the very simplest case of a predictor matrix **X** and a response matrix **Y** is known as simple linear regression and it is a problem of finding a regression coefficient **b**. However, because the size of the sampled data was smaller than the parameter of independent variables at the wavenumbers, the inverse problem to obtain **b** with 210 input parameters became an ill-conditioned problem. (b) Principal component analysis (PCA) can be used for reducing the number of parameters, finding principal components in a rotated orthogonal space which explains the majority of the variances in the measured spectrum data block. After reducing the number of parameters using PCA, a regression model can be used to obtain glucose predictions in the reduced input space as shown in (d) which is Principal Component Regression (PCR). (c) PLSR also rotates the coordinate system of the data space and computes a new component

called a latent variable, but it maximizes both the variance and the correlation between the dependent variables of the measured spectrum data block X and the reference glucose level Y. The regression with the reduced parameters is similar with PCR and is shown in (f). When the predictions by PCR and PLSR were compared, PLSR resulted in a slightly improved prediction(a MARD of PCR is 8.95 and the MARD of PLSR 8.67). Therefore, we used PLSR for the rest of the analyses. The associated references for the multivariate analysis are as follows:

1. Freitas, L. V. de & Freitas, A. Multivariate Analysis in Management, Engineering and the Sciences. (2013).

2. Mehmood, T., Liland, K. H., Snipen, L. & Sæbø, S. A review of variable selection methods in Partial Least Squares Regression. Chemometrics and Intelligent Laboratory Systems 118, 62–69 (2012).

3. Næs, T., Brockhoff, P. B. & Tomic, O. Multiple Regression, Principal Components Regression and Partial Least Squares Regression. in Statistics for Sensory and Consumer Science 227–247 (John Wiley & Sons, Ltd, 2010). doi:10.1002/9780470669181.ch15

4. de Jong, S. SIMPLS: An alternative approach to partial least squares regression. Chemometrics and Intelligent Laboratory Systems 18, 251–263 (1993).
